# Associations between High Serum Adipocyte Fatty Acid Binding Protein and First Hospitalization in Kidney Transplantation Patients: A 5-Year Follow-up Study

**DOI:** 10.3390/ijerph17207567

**Published:** 2020-10-18

**Authors:** Wei-Chen Lee, Ming-Che Lee, Ming-Chun Chen, Bang-Gee Hsu

**Affiliations:** 1 School of Medicine, Tzu-Chi University, Hualien 97004, Taiwan; 101311144@gms.tcu.edu.tw (W.-C.L.); mingche1229@gmail.com (M.-C.L.); 2 Department of Surgery, Hualien Tzu Chi Hospital, Buddhist Tzu Chi Medical Foundation, Hualien 97010, Taiwan; 3 Department of Pediatrics, Hualien Tzu Chi Hospital, Buddhist Tzu Chi Medical Foundation, Hualien 97010, Taiwan; 4 Division of Nephrology, Buddhist Tzu-Chi General Hospital, Hualien 970, Taiwan

**Keywords:** adipocyte fatty acid binding protein, hospitalization, kidney transplantation

## Abstract

Adipocyte fatty acid binding protein (A-FABP) is predictive of type 2 diabetes mellitus incidences and metabolic syndrome and is independently associated with atherosclerosis. The present study aimed to assess the association between serum A-FABP levels and future first hospitalization events in kidney transplantation (KT). We enrolled 72 KT patients from January through April 2012 and followed up on these subjects until June 2017. The first hospitalization events incidence was the primary endpoint. Using a commercially available enzyme immunoassay, serum A-FABP levels were measured from the patient’s fasting blood samples. During a median 65-month follow-up, 49 first hospitalization events occurred. KT patients with first hospitalization events had greater incidences of hypertension, diabetes, and higher serum blood urea nitrogen, creatinine, triglyceride, and A-FABP levels than those without the events. Kaplan–Meier analysis showed that the cumulative incidence of first hospitalization events was greater in the high A-FABP group than in the low A-FABP group. Multivariate Cox analysis with significant variables showed that serum A-FABP (hazard ratio = 1.012; 95% confidence interval = 1.000–1.025; *p* = 0.044) was independently associated with first hospitalization events among KT patients. The results revealed that serum A-FABP is associated with first hospitalization events in KT patients. However, further prospective studies are needed to determine the mechanisms underlying this association.

## 1. Introduction

Adipocyte fatty acid binding protein (A-FABP), an adipokine that has been implicated in lipid metabolism, lipolysis, and insulin sensitivity, can be used to predict the incidence of metabolic syndrome (MetS) and type 2 diabetes mellitus (DM). In addition to being independently associated with atherosclerosis [1,2], A-FABP is upregulated with deteriorating renal function in acute kidney dysfunction and chronic kidney disease (CKD) patients [3].

Kidney transplant (KT) is the most favorable treatment option for end-stage renal disease (ESRD) patients who would otherwise require dialysis [4]. Although ESRD patients are at a greater risk for cardiovascular disease (CVD) than the general population, successful KT leads to a reduced risk of CVD in these patients [5]. However, CVD remains the major cause of death after KT [6,7]. Studies have shown that these patients have a high prevalence of CVD risk factors before KT, and they suffer from new-onset diabetes, MetS, or coronary heart disease after KT, all of which are risk factors for allograft failure [8,9,10]. Besides, the use of immunosuppressive medications is also a risk factor, as it may induce MetS, diabetes, and coronary heart disease in KT patients [10,11].

Successful KT is closely related to the components of MetS, although the actual factors that influence first hospitalization events in KT patients remain unclear. Accumulating evidence suggests a correlation between serum A-FABP levels and renal function; thus, A-FABP is negatively correlated with estimate glomerular filtration rate (eGFR) particularly because of its glomerular and tubular handling [12]. However, thus far, no study has investigated whether the usefulness of serum A-FABP level is associated with first hospitalization events in KT patients. Therefore, the present study aimed to determine whether serum A-FABP can be used as a prognostic marker of first hospitalization events in KT patients.

## 2. Materials and Methods

### 2.1. Study Approval

The study protocol was approved by the Institutional Review Board of the Tzu Chi University and Hospital (approval no. IRB100-91), and informed consent was obtained from all participants before study participation.

### 2.2. Participants

The cohort of this cross-sectional study included 72 patients who underwent KT at the Buddhist Hualien Tzu Chi Hospital between January and April 2012 and were followed up as outpatients up to 30 June 2017. The exclusion criteria included malignancy, acute infection, previous delayed graft function, acute transplant rejection at the time of blood sampling, and declined to provide informed consent. Measurements of systolic and diastolic blood pressure (SBP and DBP, respectively) obtained three times at 5-min intervals were averaged for analysis. Hypertension was defined according to the Eighth Joint National Committee guidelines: SBP ≥ 140 mmHg and/or DBP ≥ 90 mmHg or the use of any antihypertensive drug over the past two weeks. DM was diagnosed as fasting plasma glucose levels ≥ 126 mg/dL or the use of an oral hypoglycemic medication or insulin [13,14]. All the deceased donor KT received kidney from donor after brain death. 

### 2.3. Anthropometric Analysis

With patients wearing light clothing and no shoes, their body weight (BW) and height were measured to the nearest 0.50 kg and 0.50 cm, respectively. According to the Quetelet’s formula, body mass index (BMI) was calculated as the weight in kilograms divided by the height in meters squared [15,16].

### 2.4. Biochemical Analyses

All the participants enrolled in this study received fasting blood sampling immediately after provided informed consent at outpatient’s department between January and April 2012. After overnight fasting, about 5 mL blood sample was collected from each patient and immediately centrifuged at 3000 × g for 10 min. Serum fasting glucose, albumin, triglyceride (TG), total cholesterol (TCH), high-density lipoprotein cholesterol, low-density lipoprotein cholesterol, blood urea nitrogen (BUN), and creatinine levels were measured using an auto-analyzer (COBAS Integra 800; Roche Diagnostics, Basel, Switzerland) [14]. Serum A-FABP levels were quantified using a commercially available enzyme immunoassay (SPI-BIO, Montigny le Bretonneux, France) [14,15]. We used the Modification of Diet in Renal Disease formula to eGFR.

### 2.5. First Hospitalization Event Monitoring

The primary end point was the incidence of first hospitalization events (i.e., acute kidney injury, infection, CVD, and hospitalization due to other causes). We used the last hospital outpatient or inpatient visit, the last telephone interview, or 30 June 2017 as the follow-up duration (months) estimation. Moreover, the event time (months) was calculated as the date of the first hospitalization event. All participants were followed up by a study nurse who was blinded to the study protocol and the patients’ baseline measurements.

### 2.6. Statistical Analysis

The collected data were tested for normal distribution using Kolmogorov–Smirnov test. Normally distributed data were expressed as mean ± standard deviation, and comparisons between participants were performed using the Student’s independent t-test (two tailed). Non-normally distributed data (TGs, fasting glucose, BUN, creatinine) were compared using Mann–Whitney U test and are expressed as the median and interquartile range. Variables expressed as the number of patients was analyzed using the chi-square test. Kaplan–Meier survival curves with the log-rank test were used to estimate event-free survival during the follow-up period based on the median A-FABP level. Univariate and multivariate Cox regression models were used to identify factors associated with first hospitalization events. Data were analyzed using SPSS for Windows (version 19.0; IBM Corporation, Armonk, NY, USA). A two-tailed *p*-value < 0.05 was considered significant.

## 3. Results

Demographic, biochemical and clinical information on the 72 KT patients are presented in Table 1. In total, 14 (19.4%) and 24 (33.3%) patients had DM and hypertension, respectively. Median time from treatment to first hospitalization was 81 months [interquartile range 60–121 months]. The high A-FABP group (median A-FABP level ≥ 29.05 ng/mL) had a greater female ratio (*p* = 0.033), higher BMI (*p* = 0.036) and SBP (*p* = 0.003), and elevated serum TCH (*p* = 0.014), TG (*p* = 0.040), BUN (*p* = 0.001), creatinine (*p* = 0.032), and A-FABP (*p* < 0.001) levels than the low A-FABP group (median A-FABP level < 29.05 ng/mL); however, the patients in the former were shorter in height (*p* = 0.029) and had a lower mean eGFR (*p* = 0.008). There were no significant between-group differences in comorbidity with DM or hypertension or immunosuppressive drugs used between the two groups.

By the last follow-up or 30 June 2017, 49 first hospitalization events had occurred (acute kidney injury, *n* = 22; infection, *n* = 22; CVD, *n* = 2; and hospitalization due to other causes, *n* = 4). Patients with first hospitalization events had a higher prevalence of DM (*p* = 0.027), hypertension (*p* = 0.049), and steroid used (*p* = 0.024) as well as higher serum TG (*p* = 0.040), BUN (*p* = 0.012), creatinine (*p* = 0.032), and A-FABP (*p* = 0.011) levels than those without the events (Table 2). The clinical variables of the KT patients divided by acute kidney injury related first hospitalization and infection related first hospitalization as shown in Table S1. Patients with acute kidney injury related first hospitalization events had a higher prevalence of hypertension (*p* = 0.042), steroid used (*p* = 0.038) as well as higher serum BUN (*p* < 0.001), creatinine (*p* = 0.008) and A-FABP (*p* = 0.004) levels, and lower eGFR (*p* = 0.004) than those without the events. Patients with infection related first hospitalization events had a higher prevalence of steroid used (*p* = 0.038) as well as higher serum BUN (*p* = 0.033) and A-FABP (*p* = 0.036) levels than those without the events.

**Figure 1:**
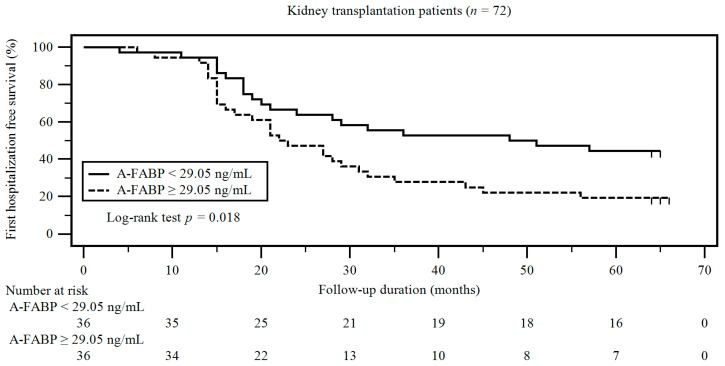
Kaplan–Meier analysis of A-FABP serums levels and first hospitalization events in KT patients.

Kaplan–Meier analysis showed that the cumulative incidence of first hospitalization events was higher in the high A-FABP group than in the low A-FABP group (log-rank *p* = 0.018) (Figure 1). The Kaplan–Meier analysis showed that the cumulative incidence of acute kidney injury related first hospitalization events was higher in the high A-FABP group than in the low A-FABP group (log-rank *p* = 0.017) (Figure S1). Furthermore, the Kaplan–Meier analysis showed that the cumulative incidence of infection related first hospitalization events was higher in the high A-FABP group than in the low A-FABP group (log-rank *p* = 0.027) (Figure S2).

The results of univariate and multivariate Cox regression analyses of correlations between serum A-FABP levels and first hospitalization events are shown in Table 3. Univariate Cox regression analysis demonstrated that serum A-FABP levels [hazard ratio (HR) = 1.018; 95% confidence interval (CI) = 1.009–1.021; *p* < 0.001] levels were positively associated with first hospitalization events in KT patients, as did multivariate Cox regression analysis adjusted for age gender, and BMI (HR = 1.019; 95% CI = 1.008–1.027; *p* = 0.001) (Model 1). Furthermore, multivariate Cox regression analysis with the variables in Model 1 plus DM and hypertension (Model 2) as well as the variables in Model 2 plus eGFR, TGs, and steroid used (Model 3) showed that serum A-FABP levels were independently associated with first hospitalization events in KT patients (Model 2: HR = 1.015; 95% CI = 1.004–1.027; *p* = 0.009; Model 3: HR = 1.012; 95% CI = 1.000–1.025; *p* = 0.044) and a 1.2% increase in the risk of hospitalization events for every 1-ng/mL elevation in A-FABP serum levels in Model 3. The results of univariate and multivariate Cox regression analyses of correlations between serum A-FABP levels and acute kidney injury related first hospitalization events and infection related first hospitalization as shown in Tables S2 and S3. Serum A-FABP levels were independently associated with acute kidney injury related first hospitalization events in KT patients (Model 3: HR = 1.023; 95% CI = 1.004–1.043; *p* = 0.020, Table S2). However, serum A-FABP levels did not has significant associated with infection related first hospitalization events in KT patients (Model 3: HR = 1.007; 95% CI = 0.988–1.027; *p* = 0.454, Table S3).

## 4. Discussion

The major findings from our study are that KT patients with first hospitalization events had high serum A-FABP levels than those without the events. The cumulative incidence of first hospitalization events was correlated with high A-FABP serum levels. Besides, A-FABP values had an independent association with first hospitalization events in KT patients, with a 1.2% increase in the risk of hospitalization events for every 1-ng/mL elevation in A-FABP serum levels.

A-FABP, an adipokine produced by adipocytes, endothelial cells, and macrophages, is a polypeptide that transports long-chain fatty acids across the adipocyte cytoplasm to subcellular compartments to regulate lipid metabolism [17,18]. Studies have demonstrated that A-FABP is crucial to lipid metabolism, insulin sensitivity, and the activation of inflammatory pathways related to obesity, MetS, type 2 DM, and atherosclerosis [1,2,14]. Besides, A-FABP serum levels have been demonstrated to be positively associated with several components of MetS, including dyslipidemia, hyperglycemia, and hypertension [19,20]. A multicenter prospective study of 578 volunteers in Spain revealed that baseline plasma A-FABP levels were associated with new-onset atherogenic dyslipidemia over a 6-year follow-up period [21]. In line with the findings of previous studies, our current study demonstrated that high circulating A-FABP levels are positively associated with BMI, SBP, TCH and TG levels, indicating a correlation between A-FABP and MetS.

In addition to the correlations with MetS, type 2 DM, and CVD, A-FABP has been reported to be positively associated with renal function in many studies [3,22,23,24]. Previous studies have found that increased A-FABP serum levels are significantly associated with mild CKD [22] and diabetic nephropathy with or without albuminuria [23,24]. Furthermore, Sommer et al., have revealed that circulating A-FABP levels are 10-fold higher in chronic hemodialysis subjects than in control patients not receiving hemodialysis [25]. In a German study published in 2014 that enrolled 532 patients with CKD and 32 patients before and within 30 h after elective unilateral nephrectomy, as a model of acute renal dysfunction, multivariate analysis revealed that A-FABP serum levels were positively associated with impaired renal function in this cohort. A-FABP levels are prominently upregulated with increasing eGFR, covering the whole spectrum of chronic kidney dysfunction. In addition, circulating A-FABP levels are reportedly significantly elevated after unilateral nephrectomy compared with the pre-surgical values (42.1 vs. 29.3 mg/L) [3]. The present study’s results similarly demonstrated that in KT patients, high A-FABP levels were positively associated with BUN and creatinine levels, but negatively associated with eGFR, compared with low A-FABP levels.

KT has become the preferred treatment option for patients with irreversible renal failure. Although noticeable improvements in allograft and patient survival have been noted in past decades, CVD-induced allograft failure and patient death remain the major causes of renal allograft loss [6,7]. Post-transplant hyperlipidemia, a risk factor for CVD-related mortality and graft loss from chronic allograft nephropathy, is reported in up to 60% of recipients [26,27]. A high prevalence (20–65%) of MetS after KT has been reported in several studies [28,29,30]. In a cross-sectional study with a mean follow-up of 6 years, MetS was found to be associated with poor graft function, and TG levels were independently associated with renal dysfunction [30]. Not surprisingly, previous studies have revealed that MetS is an important risk factor for DM [31], coronary artery disease, and stroke [32], and established risk factors for CVD promote allograft failure. Moreover, other studies have found that MetS itself may lead to renal function impairment and deterioration [33,34]. To assess the impact of MetS on KT patients, a recent meta-analysis of 1269 KT patients noted that MetS was identified as an independent risk factor for CVD-related graft loss and death [35]. The results of the present study also revealed that KT patients with first hospitalization events had higher serum TG levels and higher prevalence of DM and hypertension, the important components of MetS, than control subjects, in accordance with the pathophysiology described above.

The underlying mechanism of the development of MetS in KT patients is likely to be multifactorial. Appetite improvement after KT, as an indication of resolved uremic syndrome, and steroid use may promote BW gain, contributing to the obesity and MetS development [35]. Besides, many immunosuppressive medications commonly used after KT, such as corticosteroids, mammalian target-of-rapamycin inhibitors, and calcineurin inhibitors, also lead to the occurrence of MetS [36]. Although accumulating evidence suggests that A-FABP is correlated with renal function and it remains unclear whether A-FABP is predictive of the prognosis of KT patients. In the present study, Kaplan–Meier analysis showed that the cumulative incidence of first hospitalization events was higher in the high A-FABP group than in the low A-FABP group. Multivariate Cox regression analysis revealed that serum A-FABP levels were independently associated with first hospitalization events in KT patients. As mentioned earlier, A-FABP plays a vital role in the development of MetS and CVD, and MetS is a risk factor for CVD-related graft loss and death after KT, suggesting that high A-FABP levels are predictive of poor graft outcome in KT patients because of the aggravation of MetS components. In this study, higher A-FABP level is associated with first hospitalization events in KT patients.

Our study had some limitations. First, this study had potential selection bias and a small sample size due to it being a single-center, cross-sectional study. Therefore, further longitudinal larger-scale multicenter researches are needed to validate these results. Second, some confounders, such as smoking, were not included and may have influenced this study’s predictive power [37]. Third, the serum FABP4 levels are affected by the production of adipocytes, enhanced secretion via lipolysis and disposal via the kidney [12]. Therefore, it is difficult to evaluate adiposity composition and lipolysis condition of the patients, and unknown recovery situation of renal function after post-renal transplantation in study subjects may pose some bias in our study. Fourth, although the present study results showed that first hospitalization events of KT patients were closely related to A-FABP serum levels and the components of MetS, the risk factors of MetS may not equally contribute to the outcomes in KT patients [9,30]. However, few studies focus on the relationship between A-FABP and the outcomes among KT patients. Nevertheless, this study is the first to examine the relationship between A-FABP serum levels among KT patients with first hospitalization events.

## 5. Conclusions

Our study demonstrates that higher serum A-FABP levels are independently associated with first hospitalization events in KT patients. Further prospective research is needed to elucidate the association’s mechanisms.

## Figures and Tables

**Table 1:** The Clinical variable of the 72 kidney transplantation patients according to serum median adipocyte fatty acid binding protein levels.

Variables	All Participants	Low A-FABP Group	High A-FABP Group	*p* Value
	(*n* = 72)	(*n* = 36)	(*n* = 36)	
Age (years)	52.15 ± 9.64	51.17 ± 9.87	53.14 ± 9.44	0.389
KT duration (months)	72.90 ± 43.27	74.39 ± 49.76	71.42 ± 36.29	0.773
Height (cm)	162.31 ± 8.23	164.42 ± 7.46	160.19 ± 8.52	0.029 *
Body weight (kg)	62.85 ± 12.40	61.75 ± 10.66	63.94 ± 13.99	0.456
Body mass index (kg/m^2^)	23.81 ± 4.20	22.77 ± 3.18	24.84 ± 4.85	0.036 *
SBP (mmHg)	133.79 ± 10.43	133.47 ± 15.90	144.72 ± 15.68	0.003 *
DBP (mmHg)	86.21 ± 11.01	84.92 ± 11.13	87.50 ± 10.89	0.323
Albumin (mg/dL)	4.14 ± 0.48	4.24 ± 0.38	4.04 ± 0.55	0.082
Total cholesterol (mg/dL)	195.81 ± 45.47	182.81 ± 31.63	208.81 ± 53.38	0.014 *
Triglyceride (mg/dL)	114.50 (80.25–167.00)	95.50 (72.00–148.00)	133.50 (81.50–209.75)	0.040 *
HDL-C (mg/dL)	51.19 ± 16.09	52.36 ± 13.86	50.03 ± 18.17	0.542
LDL-C (mg/dL)	108.56 ± 39.43	109.32 ± 44.21	107.81 ± 34.63	0.872
Fasting glucose (mg/dL)	94.50 (86.50–110.00)	93.00 (85.25–99.00)	97.50 (88.00–134.75)	0.199
Blood urea nitrogen (mg/dL)	16.00 (17.00–34.75)	18.00 (14.25–25.75)	26.00 (19.50–47.00)	0.001 *
Creatinine (mg/dL)	1.60 (1.230–2.10)	1.50 (1.13–1.90)	1.85 (1.43–2.60)	0.032 *
eGFR (mL/min)	43. 74 ± 21.81	50.50 ± 21.24	36.97 ± 20.49	0.008 *
A-FABP (ng/mL)	40.53 ± 31.37	15.91 ± 7.07	65.16 ± 26.43	<0.001 *
Female, *n* (%)	33 (45.8)	12 (33.3)	21 (58.3)	0.033 *
Diabetes, *n* (%)	14 (19.4)	4 (11.1)	10 (27.8)	0.074
Hypertension, *n* (%)	24 (33.3)	9 (25.0)	15 (41.7)	0.134
Deceased donor KT, *n* (%)	63 (87.5)	34 (94.4)	29 (80.6)	0.075
Tacrolimus use, *n* (%)	43 (59.7)	23 (63.9)	20 (55.6)	0.471
Mycophenolate mofetil use, *n* (%)	53 (73.6)	26 (72.2)	27 (75.0)	0.789
Steroid use, *n* (%)	56 (77.8)	25 (69.4)	31 (86.1)	0.089
Rapamycin use, *n* (%)	14 (19.4)	6 (16.7)	8 (22.2)	0.551
Cyclosporine use, *n* (%)	16 (22.2)	6 (16.7)	10 (27.8)	0.257

Values for continuous variables given as means ± standard deviation and compared by Student’s *t*-test; variables not normally distributed given as medians and interquartile range and compared by Mann-Whitney U test; values are presented as number (%), and analysis was performed using the chi-square test. A-FABP, adipocyte fatty acid binding protein; DBP, diastolic blood pressure; eGFR, estimated glomerular filtration rate; HDL-C, high-density lipoprotein cholesterol; KT, kidney transplantation; LDL-C, low-density lipoprotein cholesterol; SBP, systolic blood pressure. * *p* < 0.05 was considered statistically significant.

**Table 2:** Clinical variables of the kidney transplantation with or without first hospitalization events.

Variables	Participants without Hospitalization Events	Participants with Hospitalization Events	*p* Value
	(*n* = 23)	(*n* = 49)	
Age (years)	50.57 ± 8.10	52.90 ± 10.27	0.342
KT duration (months)	69.13 ± 40.65	74.67 ± 44.74	0.616
Height (cm)	162.83 ± 8.27	162.06 ± 8.29	0.716
Body weight (kg)	61.26 ± 11.92	63.59 ± 12.66	0.461
Body mass index (kg/m^2^)	23.00 ± 3.49	24.19 ± 4.48	0.265
SBP (mmHg)	131.65 ± 10.03	134.80 ± 10.57	0.236
DBP (mmHg)	84.70 ± 10.43	86.92 ± 11.30	0.428
Albumin (mg/dL)	4.23 ± 0.34	4.10 ± 0.53	0.294
Total cholesterol (mg/dL)	185.13 ± 36.20	200.82 ± 48.78	0.174
Triglyceride (mg/dL)	102.00 (77.00–142.00)	135.00 (80.50–195.00)	0.040 *
HDL-C (mg/dL)	51.17 ± 12.55	51.20 ± 17.63	0.994
LDL-C (mg/dL)	107.00 ± 25.76	109.30 ± 44.65	0.820
Fasting glucose (mg/dL)	93.00 (85.00–99.00)	96.00 (88.00–135.50)	0.199
Blood urea nitrogen (mg/dL)	18.00 (14.00–23.00)	26.00 (18.00–40.50)	0.012 *
Creatinine (mg/dL)	1.40 (1.00–2.10)	1.80 (1.35–2.10)	0.032 *
eGFR (mL/min)	51.04 ± 25.21	40.31 ± 19.36	0.051
A-FABP (ng/mL)	26.90 ± 22.69	46.93 ± 32.99	0.011 *
Female, *n* (%)	9 (39.1)	24 (49.0)	0.434
Diabetes, *n* (%)	1 (4.3)	13 (19.6)	0.027 *
Hypertension, *n* (%)	4 (17.4)	20 (40.8)	0.049 *
Deceased donor KT, *n* (%)	19 (82.6)	44 (89.8)	0.390
Tacrolimus use, *n* (%)	15 (65.2)	28 (57.1)	0.515
Mycophenolate mofetil use, *n* (%)	19 (82.6)	34 (69.4)	0.235
Steroid use, *n* (%)	15 (65.2)	43 (87.8)	0.024 *
Rapamycin use, *n* (%)	2 (8.7)	12 (24.5)	0.114
Cyclosporine use, *n* (%)	6 (26.1)	10 (20.4)	0.589

Values for continuous variables given as means ± standard deviation and compared by Student’s *t*-test; variables not normally distributed given as medians and interquartile range and compared by Mann–Whitney U test; values are presented as number (%), and analysis was performed using the chi-square test. A-FABP, adipocyte fatty acid binding protein; DBP, diastolic blood pressure; eGFR, estimated glomerular filtration rate; HDL-C, high-density lipoprotein cholesterol; KT, kidney transplantation; LDL-C, low-density lipoprotein cholesterol; SBP, systolic blood pressure. * *p* < 0.05 was considered statistically significant.

**Table 3:** Cox regression for first hospitalization events of adipocyte fatty acid binding protein levels among the 72 kidney transplantation patients.

	Unadjusted		Model 1		Model 2		Model 3	
	HR (95% CI)	*p* Value	HR (95% CI)	*p* Value	HR (95% CI)	*p* Value	HR (95% CI)	*p* Value
A-FABP, 1 ng/mL	1.018	<0.001 *	1.019	0.001 *	1.015	0.009 *	1.012	0.044 *
	(1.009–1.021)		(1.008–1.027)		(1.004–1.027)		(1.000–1.025)	

Model 1 is adjusted for age, gender and body mass index. Model 2 is adjusted for the Model 1 variables and for diabetes mellitus and hypertension. Model 3 is adjusted for the Model 2 variables and for glomerular filtration rate, triglyceride and steroid used. * *p* < 0.05 was considered statistically significant.

## Supplementary Materials

The following are available online at https://www.mdpi.com/1660-4601/17/20/7567/s1, Figure S1: Kaplan–Meier analysis of A-FABP serums levels and acute kidney injury related first hospitalization events in KT patients, Figure S2: Kaplan–Meier analysis of A-FABP serums levels and infection related first hospitalization events in KT patients, Table S1: Clinical variables of the kidney transplantation with or without acute kidney injury or infection related first hospitalization events, Table S2. Cox regression for acute kidney injury related first hospitalization events of A-FABP levels among the 45 kidney transplantation patients, Table S3. Cox regression for infection related first hospitalization events of A-FABP levels among the 45 kidney transplantation patients.
