# Supplementary material for: Associations between High Serum Adipocyte Fatty Acid Binding Protein and First Hospitalization in Kidney Transplantation Patients: A 5-Year Follow-up Study
Source: Int J Environ Res Public Health. 2020 Oct 18;17(20):7567. doi: 10.3390/ijerph17207567 (PMC7589115; doi:10.3390/ijerph17207567)
Supplement: Supplementary file 1 [file ijerph-17-07567-s001.pdf]

Table S1. Clinical variables of the kidney transplantation with or without acute kidney injury or infection related first hospitalization events.

Variables	Participants without Hospitalization Events (*n* = 23)	Participants with AKI Related Hospitalization events (*n* = 22)	*p* Value	Participants with Infection Related Hospitalization Events (*n* = 22)	*p* Value
Age (years)	50.57 ± 8.10	53.41 ± 8.73	0.263	52.00 ± 11.45	0.629
KT duration (months)	69.13 ± 40.65	86.27 ± 50.58	0.216	60.95 ± 33.71	0.468
Height (cm)	162.83 ± 8.27	163.00 ± 7.85	0.943	159.55 ± 7.93	0.182
Body weight (kg)	61.26 ± 11.92	63.50 ± 12.92	0.549	63.05 ± 12.57	0.627
Body mass index (kg/m^2)	23.00 ± 3.49	23.87 ± 4.44	0.464	24.77 ± 4.86	0.165
SBP (mmHg)	131.65 ± 10.03	136.64 ± 7.86	0.071	133.68 ± 12.51	0.551
DBP (mmHg)	84.70 ± 10.43	87.18 ± 11.61	0.451	87.09 ± 11.64	0.471
Albumin (mg/dL)	4.23 ± 0.34	4.11 ± 0.43	0.294	4.16 ± 0.54	0.619
Total cholesterol (mg/dL)	185.13 ± 36.20	211.00 ± 55.78	0.071	193.18 ± 41.22	0.490
Triglyceride (mg/dL)	102.00 (77.00–142.00)	107.50 (82.50–176.75)	0.407	140.50 (73.50–212.50)	0.156
HDL-C (mg/dL)	51.17 ± 12.55	52.05 ± 19.40	0.858	50.64 ± 16.76	0.903
LDL-C (mg/dL)	107.00 ± 25.76	99.91 ± 41.58	0.493	114.95 ± 25.85	0.307
Fasting glucose (mg/dL)	93.00 (85.00–99.00)	88.00 (88.00–116.50)	0.419	93.00 (83.00–123.50)	0.716
Blood urea nitrogen (mg/dL)	18.00 (14.00–23.00)	29.50 (22.75–47.50)	<0.001 *	25.00 (17.75–37.75)	0.033 *
Creatinine (mg/dL)	1.40 (1.00–2.10)	1.85 (1.56–2.73)	0.008 *	1.80 (1.10–2.13)	0.562
eGFR (mL/min)	51.04 ± 25.21	32.45 ± 14.32	0.004 *	44.86 ± 21.71	0.384
A-FABP (ng/mL)	26.90 ± 22.69	54.15 ± 35.51	0.004 *	44.70 ± 31.82	0.036 *
Female, *n* (%)	9 (39.1)	10 (45.5)	0.668	12 (54.5)	0.300

Diabetes, *n* (%)	1 (4.3)	5 (22.7)	0.070	5 (22.7)	0.070
Hypertension, *n* (%)	4 (17.4)	10 (45.5)	0.042 *	9 (40.9)	0.082
Deceased donor KT, *n* (%)	19 (82.6)	20 (90.9)	0.413	19 (86.4)	0.728
Tacrolimus use, *n* (%)	15 (65.2)	11 (54.0)	0.302	14 (63.6)	0.912
Mycophenolate mofetil use, *n* (%)	19 (82.6)	15 (68.2)	0.260	15 (68.2)	0.260
Steroid use, *n* (%)	15 (65.2)	20 (90.9)	0.038 *	20 (90.9)	0.038 *
Rapamycin use, *n* (%)	2 (8.7)	6 (27.3)	0.103	5 (22.7)	0.194
Cyclosporine use, *n* (%)	6 (26.1)	6 (27.3)	0.928	4 (18.2)	0.524

Values for continuous variables given as means ± standard deviation and compared by Student's *t*-test; variables not normally distributed given as medians and interquartile range and compared by Mann-Whitney U test; values are presented as number (%), and analysis was performed using the chi-square test. AKI, acute kidney injury; A-FABP, adipocyte fatty acid binding protein; DBP, diastolic blood pressure; eGFR, estimated glomerular filtration rate; HDL-C, high-density lipoprotein cholesterol; KT, kidney transplantation; LDL-C, low-density lipoprotein cholesterol; SBP, systolic blood pressure. * $p < 0.05$ was considered statistically significant.

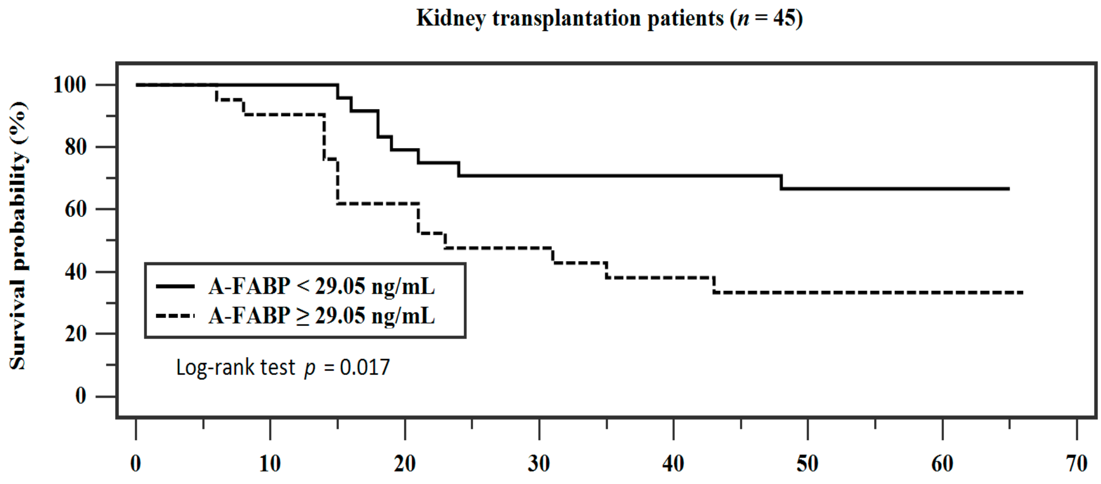

Follow-up duration (months)

Number at risk							
**Group: A-FABP < 29.05 ng/mL**							
**24**	**24**	**19**	**17**	**17**	**16**	**16**	**0**
**Group: A-FABP ≥ 29.05 ng/mL**							
**21**	**19**	**13**	**10**	**8**	**7**	**7**	**0**

Figure S1. Kaplan–Meier analysis of A-FABP serums levels and acute kidney injury related first hospitalization events in KT patients.

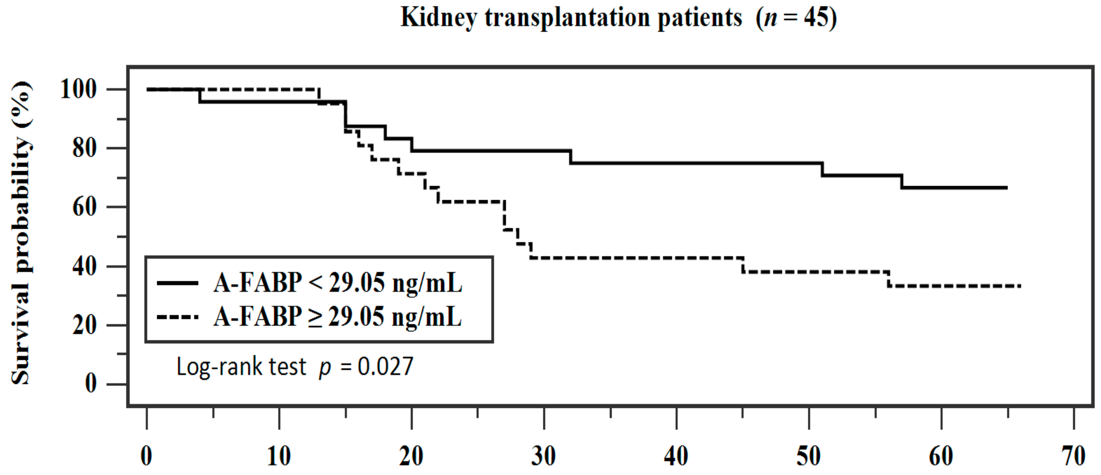

Follow-up duration (months)

Number at risk							
**Group: A-FABP < 29.05 ng/mL**							
**24**	**23**	**19**	**19**	**18**	**18**	**16**	**0**
**Group: A-FABP ≥ 29.05 ng/mL**							
**21**	**21**	**15**	**9**	**9**	**8**	**7**	**0**

Figure S2. Kaplan–Meier analysis of A-FABP serums levels and infection related first hospitalization events in KT patients.

Table S2. Cox regression for acute kidney injury related first hospitalization events of adipocyte fatty acid binding protein levels among the 45 kidney transplantation patients.

	Unadjusted		Model 1		Model 2		Model 3	
	**HR (95% CI)**	***p* Value**	**HR (95% CI)**	***p* Value**	**HR (95% CI)**	***p* Value**	**HR (95% CI)**	***p* Value**
A-FABP, 1 ng/mL	1.027 (1.013–1.041)	<0.001 *	1.032 (1.015–1.049)	<0.001 *	1.027 (1.009–1.046)	0.003 *	1.023 (1.004–1.043)	0.020 *

Model 1 is adjusted for age, gender and body mass index. Model 2 is adjusted for the Model 1 variables and for diabetes mellitus and hypertension. Model 3 is adjusted for the Model 2 variables and for glomerular filtration rate, triglyceride and steroid used. * $p < 0.05$ was considered statistically significant.

Table S3. Cox regression for infection related first hospitalization events of adipocyte fatty acid binding protein levels among the 45 kidney transplantation patients.

	Unadjusted		Model 1		Model 2		Model 3	
	**HR (95% CI)**	***p* Value**	**HR (95% CI)**	***p* Value**	**HR (95% CI)**	***p* Value**	**HR (95% CI)**	***p* Value**
A-FABP, 1 ng/mL	1.017 (1.004–1.030)	0.013 *	1.014 (0.999–1.030)	0.075	1.010 (0.992–1.028)	0.281	1.007 (0.988–1.027)	0.454

Model 1 is adjusted for age, gender and body mass index. Model 2 is adjusted for the Model 1 variables and for diabetes mellitus and hypertension. Model 3 is adjusted for the Model 2 variables and for glomerular filtration rate, triglyceride and steroid used. * $p < 0.05$ was considered statistically significant.
